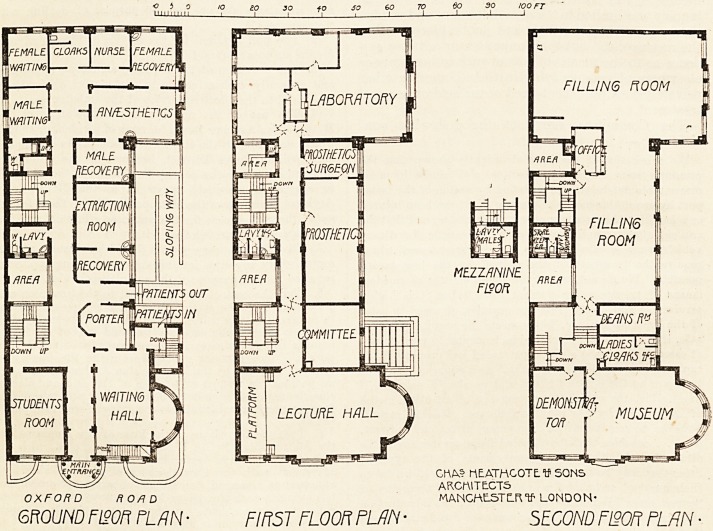# The Dental Hospital of Manchester

**Published:** 1909-04-03

**Authors:** 


### THE DENTAL HOSPITAL OF MANCHESTER.

This hospital was founded in 1884 with the double purpose of establishing a much needed charity and of founding a fully-equipped school for the training of dental surgeons. For the latter purpose it is associated with the University of Manchester, and also with the Royal Infirmary; and affords the necessary training for students preparing for the L.D.S. and B.D.S. qualifications of the University, the examinations for which are held in Manchester; also for the L.D.S. qualification of the Royal College of Surgeons of England, and of other licensing bodies. The necessary instruction is also provided for students who, having served an apprenticeship in dental mechanics with a registered dental surgeon, take the final courses of their professional training at the hospital.

The new building occupies a corner site in the Oxford Road, and is lighted fully on three sides and partially on the fourth; the site therefore would appear to be admirably suited for the purposes of the hospital in the matter of light, so all-important for dental work. The entrance and exit for patients are on the ground floor, and are separated by the porters' office, which thus commands both. A large waiting-hall adjoins the patients' entrance; on one side of this hall is a large curved bay window, separated from the rest by a glazed screen, the precise object of which is not apparent. A staircase leading out of the waiting-room is, we presume, for access to the sanitary offices. The department for extraction is on this floor, and comprises a room for ordinary extraction (*i.e.* without anæsthesia), with a recovery-room adjoining; a room for extraction under anæsthesia, with separate waiting and recovery-rooms, for male and female patients, lavatories for each sex, and a room for the nurse. Two staircases, and a common room for students complete the accommodation on this floor. Cloak-rooms, bicycle-rooms, and lavatories for students are provided in the basement—and a sloping way in the front area affords access to the bicycle-room.

On the first floor is the department of prosthetics, a somewhat awkward Greek term, under which is included the mechanical work which forms so important a part of dental surgery. There is a large laboratory with bench accommodation for 50 students, with a separate laboratory for advanced students, and a private room for the surgeon in attendance. On this floor also are a committee room and a lecture hall. The "conservation," or filling department, is on the second floor, and consists of one large room with an office. Accommodation is here provided for more than 50 chairs, each of which has a standard for cabinet, bracket table, electric motor, light, and spittoon, with hot and cold water and salivary ejector. A steriliser for instruments and students' cupboards are also provided. On this floor also is a room set apart for the instruction of new students, and a museum. Lavatory accommodation for patients of both sexes is provided on this floor and on a mezzanine. The hospital is lighted throughout with electric light, and is provided with a power current for lathes, engines, and dental furnaces. The building strikes us as remarkably well planned and well lighted in every part, and reflects much credit on the architects, Messrs. Charles Heathcote and Sons, of Manchester.

## Figures and Tables

**Figure f1:**